# Linear Accelerator-Based Stereotactic Radiosurgery (SRS) for Arteriovenous Malformation (AVM) With Functional Magnetic Resonance Imaging (fMRI) Brain Mapping for Toxic Avoidance

**DOI:** 10.7759/cureus.85816

**Published:** 2025-06-11

**Authors:** Jafri M Abdullah, Gokula Kumar Appalanaido, Nur Asma Sapiai, Hazim Omar, Reduan Abdullah, Jasmin Jalil, Aini Ismafairus Abd Hamid

**Affiliations:** 1 Brain Behaviour Cluster and Department of Neurosciences, Universiti Sains Malaysia Health Campus/School of Medical Sciences, Universiti Sains Malaysia Health Campus, Kubang Kerian, MYS; 2 Advanced Medical and Dental Institute, Universiti Sains Malaysia, Penang, MYS; 3 Department of Radiotherapy, Universiti Sains Malaysia School of Medical Sciences, Kubang Kerian, MYS; 4 Department of Radiology, School of Medical Sciences, Universiti Sains Malaysia, Kubang Kerian, MYS; 5 School of Health Sciences, Universiti Sains Malaysia, Kubang Kerian, MYS

**Keywords:** arteriovenous malformation (avm), avm srs dose, brain arteriovenous malformations, functional mri (fmri), linac-based srs, treatment planning system (tps), vibrotactile areas

## Abstract

While the treatment of unruptured arteriovenous malformation of the brain (bAVM) is controversial, active intervention is usually recommended for patients who have had a previous intracerebral hemorrhage. Stereotactic radiosurgery (SRS) is the preferred treatment for bAVM when the patient is unsuitable for surgery or embolization. Currently, linear accelerator-based cranial SRS is gaining popularity, and this case report describes the multidisciplinary approach in treating a military sniper with a 3.2 cm x 3.5 cm x 3.7 cm Spetzler-Martin Grade 3 left high parietal AVM using this technique.

Functional MRI (fMRI) with block designs was used to identify the motor and vibrotactile areas of the hand and fingers. The patient underwent CT-simulation with frameless double-shell thermoplastic stereotactic mask immobilization followed by CT angiogram in the same position. After co-registration of CT simulation and MRI images, SRS planning was performed with the Eclipse Treatment Planning System (TPS). Two prescription dose levels of 20 Gy and 14 Gy were used. The dose constraints applied in the TPS were: Maximum dose (Dmax) of 16 Gray to the inner/medial wall and 12 Gray to the outer/lateral wall of the left lateral sinus, and 20 Gray to the motor and sensory regions of the brain. Radiological assessment after 32 months showed complete obliteration of the AVM, and the patient did not have any significant toxicity. He is back to his regular job as an army sniper in the armed forces.

With the current technological advent of linear accelerators and the TPS algorithms with inverse planning capacity, highly variable and conformal brain SRS plans can be generated based on an individual patient’s disease anatomy. Since bAVM is a benign disease and patients may have a long survival after treatment, it is important to ensure that the toxicity from the intervention is minimized. A multidisciplinary team successfully mapped adjacent brain areas, identified the bAVM target, and executed a complex radiotherapy plan, resulting in good outcomes for a patient with parietal bAVM near the hand grip region and left lateral sinus.

## Introduction

Arteriovenous malformation of the brain (bAVM) is a congenital defect of the vascular system that is present at birth but typically detected later in life when symptoms manifest. The reported incidence of bAVMs is 1 in 100,000; however, the prevalence is likely much higher, estimated at 18 per 100,000 population. Most bAVMs are asymptomatic, and in two-thirds of patients, intracranial bleeding is the first presentation. The reported risk factors for bAVM rupture include large size, presence of an aneurysm, venous stenosis, and exclusively deep draining or single draining vein [1]. The estimated risk of bleeding in asymptomatic individuals with bAVMs is approximately 2% per year, while for a patient who has experienced an intracranial bleed, the risk of a second bleed from the same bAVM can be as high as 18% within one year. Around 20% of patients with bAVMs also present with seizures [2].

Management of unruptured bAVMs is controversial, whether for active intervention or observation. The ARUBA randomized controlled trial showed that patients in the intervention group had more than three times the risk of stroke or death compared to those in the medical management group alone. However, this trial has its limitations in terms of very short follow-up and a high proportion of patients undergoing embolization [3]. Nevertheless, for patients who have had a previous intracerebral hemorrhage, most treating physicians will recommend active intervention to prevent a second bleed [4].

In the definitive treatment of bAVMs, the aim should be complete obliteration of the bAVM, as partial obliteration of the nidus appears to increase the risk of hemorrhage [5]. Stereotactic radiosurgery (SRS) using a high-energy photon beam is a non-invasive treatment for bAVMs with obliteration rates of 70 - 80% [6,7]. While traditionally SRS was delivered using rigid frame fixation and the gamma knife radiosurgery system (GKS) with cobalt sources, linac-based radiosurgery has now gained popularity. SRS is the preferred treatment if the bAVM is considered too risky for surgery or the patient is unfit for surgery. While the GKS uses a multiple isocentric technique to achieve good dose conformality, linac-based SRS has the advantage of an advanced computerized treatment planning system (TPS) that can “curve” the isodose lines according to the shape of the target. This is an important feature since some bAVMs are not spherical in shape, unlike metastatic brain lesions.

Despite the advancements in treatment delivery technology, identification of important Broadman’s areas in the brain is important for formulating an SRS plan that avoids high-radiation doses to the areas that control specific functions. Functional MRI (fMRI) with block designs can be used for continuous stimulus paradigms, due to their robustness in eliciting cognitive engagement and facilitating brain activation localization [8]. This case report is about the collaboration of a multidisciplinary team consisting of neurosurgeons, neuroradiologists, and radiation oncologists in identifying the bAVM, brain motor, and vibrotactile areas of the hands and fingers with fMRI and executing SRS treatment for a military sniper with previously bAVM rupture and bleed.

## Case presentation

**Figure 1:**
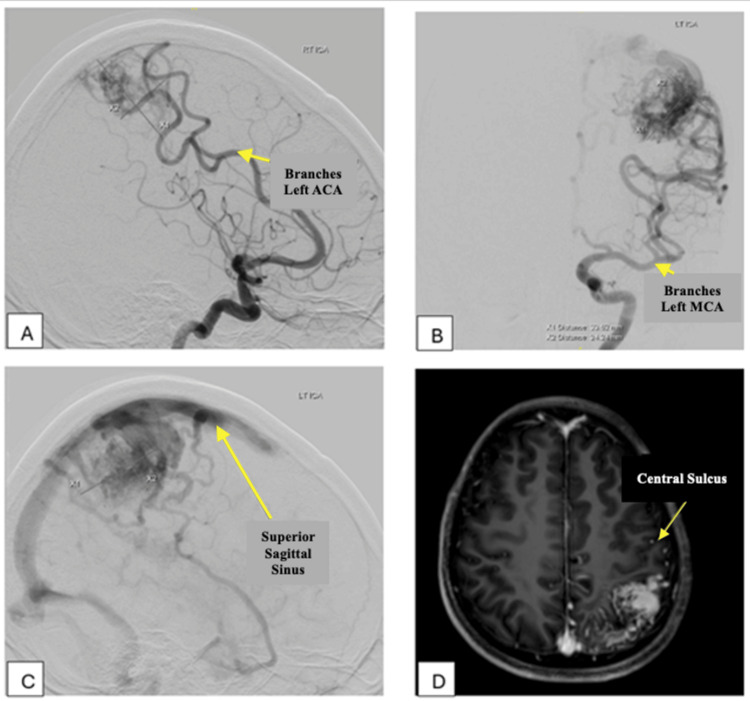
(A–C) DSA Brain and (D) MRI Brain show Spletzer Martin Grade 3 bAVM (D) evidenced by a nidus size of more than 3cm, eloquent region over postcentral gyrus and (C) cortical venous drainage into SSS. (A & B) Arterial supplies to the nidus via branches from the left ACA, MCA, and PCA. SSS: Superior sagittal sinus; ACA: anterior cerebral artery; PCA: posterior cerebral artery; MCA: middle cerebral artery

The patient presented with generalized tonic-clonic seizure that lasted around 10 minutes, accompanied by the up-rolling of the eyeballs and drooling of saliva in November 2021. Postictally, there was drowsiness, which resolved within 10 to 15 minutes. There was no history of trauma or raised intracranial pressure symptoms. Clinically, the patient was alert with a full Glasgow Coma Scale (GCS) score and no neurological deficits. Subsequent imaging with computed tomography (CT) brain, magnetic resonance imaging (MRI) Brain, and digital subtraction angiography revealed a left high parietal bAVM of Spetzler-Martin Grade 3. The nidus measured 3.2 cm x 3.5 cm x 3.7 cm at the left postcentral gyrus, with feeding arteries supplied from branches of the left middle cerebral artery (MCA), left posterior cerebral artery (PCA), and left anterior cerebral artery (ACA) with drainage into the superior sagittal sinus (SSS), as shown in Figure 1. After a multidisciplinary team discussion, it was concluded that SRS would be the best treatment option for this patient to achieve obliteration of the bAVM and preserve his crucial function in the right hand as a military sniper.

MRI-based identification of brain motor and vibrotactile areas of the hands and fingers

Blood oxygen level-dependent (BOLD) fMRI data were acquired using a T2-weighted gradient echo planar imaging sequence to investigate brain activation during two distinct tasks: (i) a self-paced handgrip task and (ii) a vibrotactile stimulation task.

**Figure 2:**
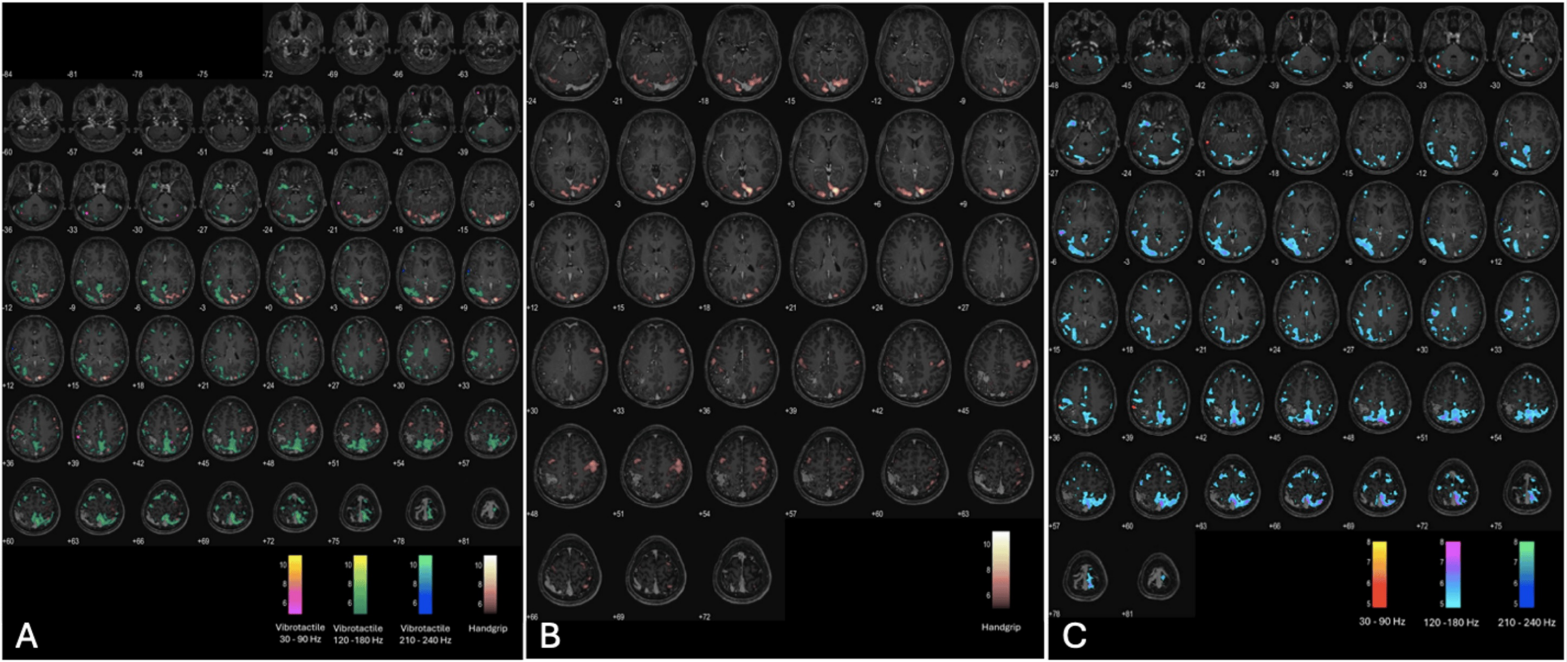
(A) Axial plane fMRI activation map with activated clusters indicating a significant increase in the blood oxygen level-dependent (BOLD) signal for (B) self-paced handgrip task and (C) vibrotactile stimulation (30-240 Hz).

For the self-paced handgrip task, the patient performed finger-tapping activities using the right hand, left hand, or both hands. These activities were guided by visual stimuli displayed on a screen and reflected onto a mirror mounted on the head coil. Each stimulus image was presented for 20 seconds. Experimental sessions consisted of 18 stimulus blocks and 17 rest blocks, alternately arranged to form a total of 35 blocks (Figures 2A, 2B). The task lasted approximately 13 minutes and was conducted using E-Prime 1.0 software (Psychology Software Tools, Inc.). Functional scans used an echo-planar imaging (EPI) series with parameters TR/TE/slice thickness/flip angle/FOV = 2000 ms/30 ms/3 mm/78°/220 x 220 mm.

For the vibrotactile stimulation task, stimuli were delivered to the right index finger using an MRI-compatible piezoelectric device (Ben Krasnow, Redwood City, CA). A block design paradigm was employed, comprising six cycles of 330-second runs, each with 16 active and 16 rest blocks. Low-frequency stimulation (30-240 Hz) was applied during active blocks (Figures 2A, 2C). Stimulus presentation and imaging acquisition were synchronized via trigger pulses from MATLAB R2021b (Mathworks Inc., Natick, MA, USA). Functional imaging for the vibrotactile task employed a separate EPI sequence with parameters TR/TE/slice thickness/flip angle/FOV = 3000 ms/33 ms/4 mm/80°/230 mm.

Additional neuroimaging data were acquired using a 3.0 T MRI scanner (Achieva, Philips, Netherlands) equipped with a 32-channel SENSE head coil. High-resolution structural images were obtained using a T1-weighted imaging sequence (TR/TE/slice thickness/FOV = 9.7 ms/4.6 ms/1.2 mm/250 mm x 250 mm) for anatomical localization (Figure 2).

CT simulation, bAVM identification, and image fusion with fMRI

**Figure 3:**
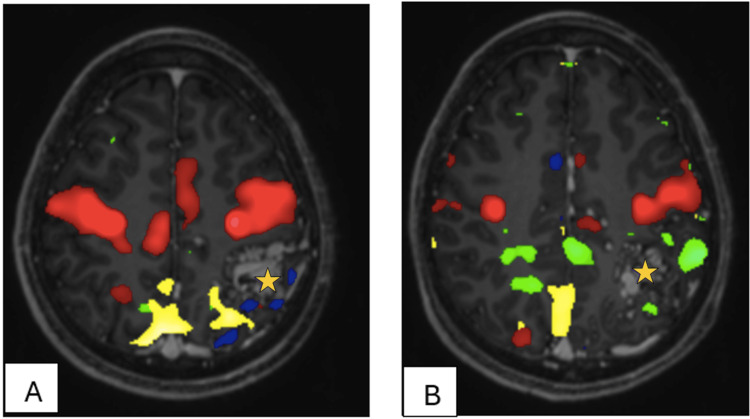
(A & B) Functional MRI of the patient while patient doing handgrip and vibrotactile stimuli of the fingertips for both hands to assess sensorimotor of bilateral upper limbs. The nidus (light orange star) is surrounded anteriorly by the motor region (red) and laterally, medially and posteriorly by the sensory region (blue, yellow and green).

**Figure 4:**
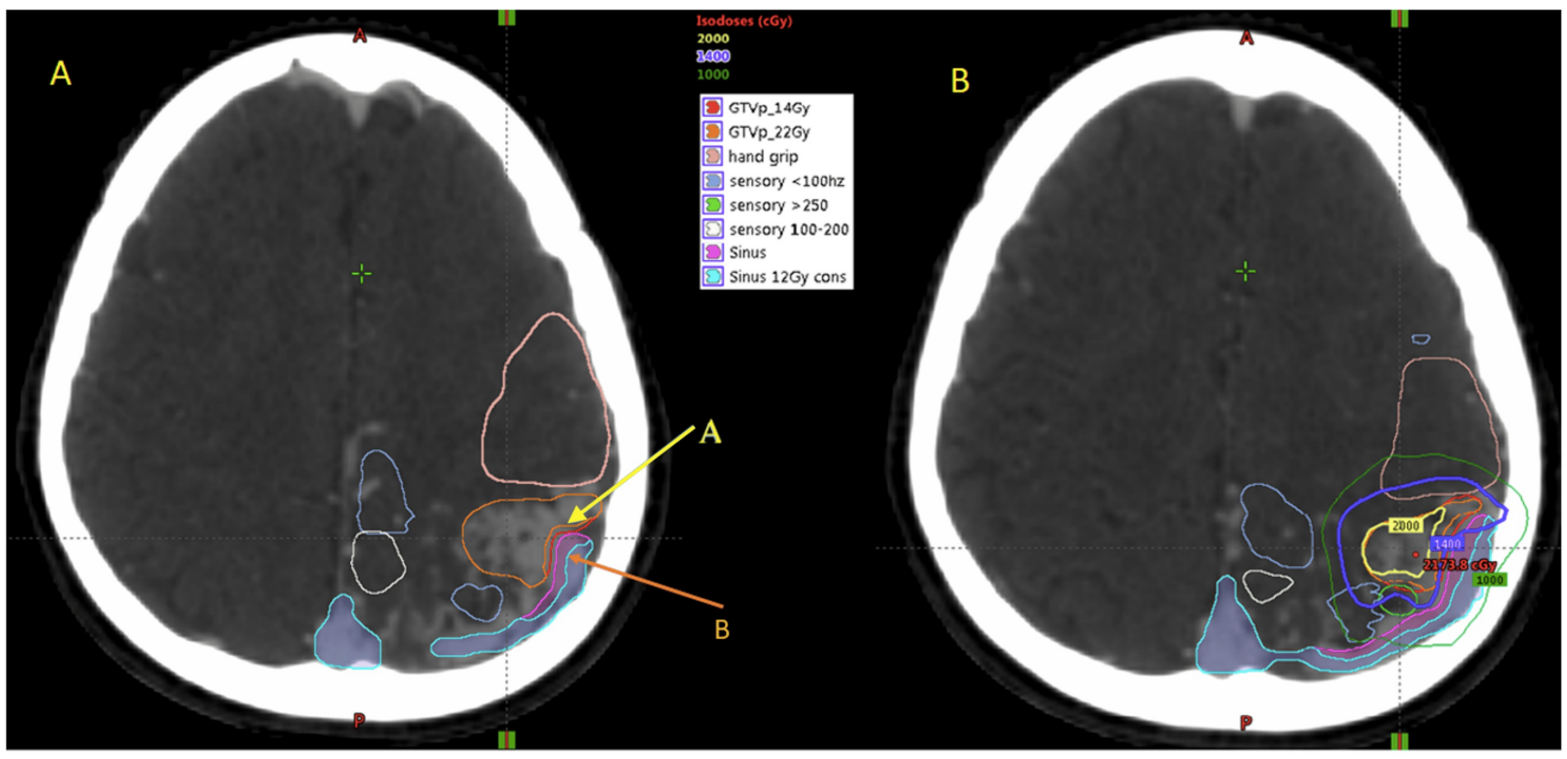
Contours on axial CT images after co-registration with MRI and CT angiogram. (A) Co-registered contours with two different prescription isodoses for the GTV (arrow A) and two different constraints for lateral sinus (arrow B). (B) Isodose lines generated by the treatment planning system showing a 14Gy isodose line (blue) covering the full GTV and 20Gy isodose line (yellow) covering the GTV with safety margin from lateral sinus.

Axial CT simulation images with a 2 mm slice thickness were acquired using the Aquillion LB CT simulator (Toshiba Medical System Corporation, Tochigi, Japan), with the patient immobilized in a frameless double-shell thermoplastic stereotactic mask (DSTSM). A CT angiogram was performed in the same position, maintaining immobilization with the DSTSM. The nidus was identified on both the CT angiogram and MRI images (Figure 3). Subsequently, the MRI, fMRI, and CTA images were fused with the CT simulation images to delineate the target and the surrounding eloquent areas for SRS planning (Figure 4). 

Radiotherapy planning and treatment delivery

Stereotactic treatment planning was performed using the Eclipse Treatment Planning System (TPS, Version 13.6; Varian Medical Systems, Inc., Palo Alto, CA, USA). Simultaneous integrated boost with volume-based prescription of two dose levels using the VMAT technique was planned: 20 Gy to be delivered to the bAVM, and a lower isodose prescription of 14 Gy to the areas adjacent to the lateral sinus (Figure 4). The dose constraint for the organ at risk (OAR) was specified as follows: maximum dose (Dmax) of 16 Gy to the inner/medial wall and 12 Gray to the outer/lateral wall of the left lateral sinus, and 20 Gray to the motor and sensory regions. The dosimetric characteristics of the treatment plan generated by the Eclipse treatment planning system are shown in Table 1. The patient was subsequently treated on A 5 mm leaf size Varian Clinac iX linear accelerator (Varian Medical Systems, Inc., Palo Alto, CA, USA) in a single fraction.

**Table 1:** Dosimetric characteristics of the Eclipse treatment plan. GTVp: gross tumor volume; Dmin/Dmean/Dmax: dose minimum/mean/maximum; D0.2cc: dose to 0.2cc; D100%/D99%/D95% / D90%: minimum dose to GTVp volume (%); CI: dose conformity index; HI: dose heterogeneity index; ccm: cubic centimeter; cGy: centi Gray

Structure	Total volume (ccm)	Dmin (cGy)	Dmean (cGy)	Dmax (cGy)	D0.2cc (cGy)	D100% (cGy)	D99% (cGy)	D95% (cGy)	D90% (cGy)	CI	HI
GTVp_14Gy	14.80	944.50	2041.00	2208.90	-	937.33	1672.69	1972.84	1998.14	0.82	0.16
GTVp_22Gy	14.50	962.50	2208.90	2208.90	-	963.49	1787.50	1983.55	2000.85	0.83	0.12
Hand grip	15.70	172.40	830.80	1989.90	1755.50	-	-	-	-	-	-
Sensory <100Hz	6.90	167.80	748.80	2121.50	1685.74	-	-	-	-	-	-
Sensory 100 - 200Hz	6.80	169.90	930.90	1987.20	1825.25	-	-	-	-	-	-
Sensory >200Hz	3.00	507.30	1179.20	2015.90	1780.20	-	-	-	-	-	-
Sinus	10.30	49.20	451.20	1883.90	1477.93	-	-	-	-	-	-
Sinus 12Gy	9.60	49.20	389.30	1811.80	1339.87	-	-	-	-	-	-

Follow-up and assessment

**Figure 5:**
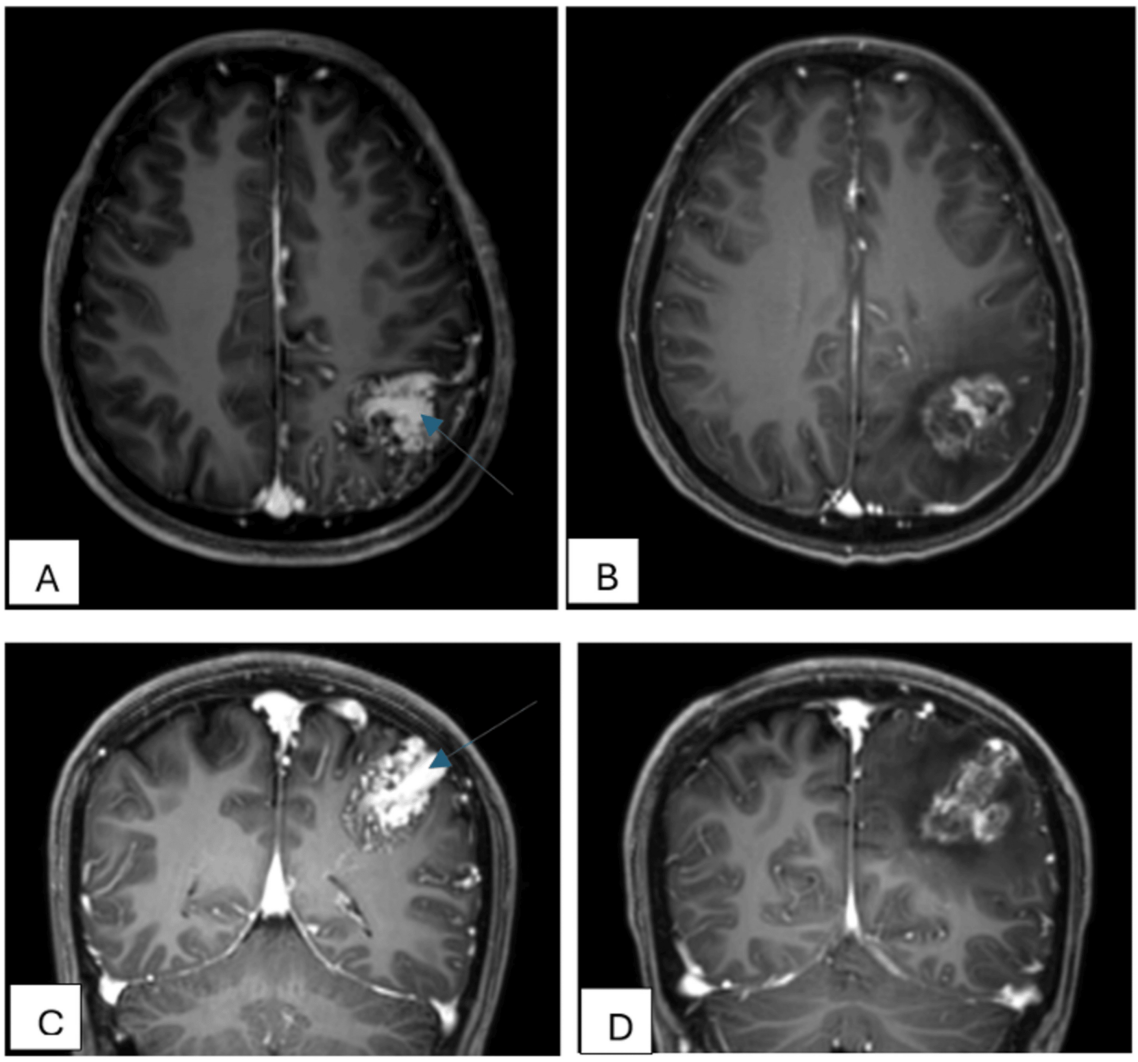
MRI brain image pre-SRS in (A) axial and (C) coronal showing nidus with large feeding arteries (blue arrow) as comparison with images of MRI brain two years after intervention (SRS) on (B & D) showing obliteration of the feeding arteries (not seen). MRI images post-intervention also show the necrosis changes of the nidus from the radiation given with regions of perilesional oedema. SRS: Stereotactic radiosurgery

The patient tolerated the treatment well, and reassessment at three months revealed right hemiparesis with motor power Medical Research Council (MRC) grading of 3, which completely resolved with intensive physiotherapy and rehabilitation. During the most recent assessment in August 2024, the nidus was stable on surveillance MRI with obliteration of feeding arteries. There was also evidence of radiation necrosis around the nidus with mild perilesional oedema, as shown in Figure 5. Clinically, the patient has no sensory or motor function deficit and intact cranial nerves’ function. The patient is currently independent in his activities of daily living (ADL) and is back to his duty as a sniper with the Armed Forces.

## Discussion

Given that this patient had a previous bleed, he is classified as high risk for rebleeding from his bAVM [4]. Consequently, our multidisciplinary team reached a consensus for active intervention. In this patient, embolization or surgery was not preferred due to the eloquent location of the bAVM [9]. fMRI-based neuroanatomical mapping revealed that the transverse sinus wall was in contact with the bAVM, while the motor cortex responsible for hand grip function was located anteriorly.

In this patient, we utilized a broad frequency range to overcome limitations in prior somatosensory research that used narrower ranges [10]. With the use of multiple frequencies in sensory mapping fMRI, we were able to selectively activate different populations of mechanoreceptors in the skin for a detailed and comprehensive map of somatosensory function and also help to differentiate the roles of different brain regions. The index finger was selected as the stimulation site due to its high mechanoreceptor density, which is essential for fine tactile discrimination [11]. While mechanoreceptor density varies across fingertips [12], the index finger provides a representative area for investigating tactile processing, particularly given the enhanced sensitivity to texture and shape in the central fingertip region.

The supramarginal gyrus (30-90 Hz stimulation), located in the parietal lobe, is an essential component for processing tactile inputs [13], specifically in perceiving the shape, size, and texture of objects through touch.

The 120-180 Hz stimulation elicited a more widespread activation pattern involving regions associated with higher-order cognitive processes with involvement in visuospatial processing and attention, while the temporal cortex may reflect experienced somatosensory processing and vibrotactile frequency discrimination [14]. Activation of the left inferior parietal gyrus, left middle frontal gyrus, and left precentral gyrus highlights the roles of sensorimotor integration and motor planning in processing stimuli within this frequency range [15].

Left postcentral gyrus in response to 210-240 Hz stimulation located in the parietal lobe, is the primary location of the somatosensory cortex (S1) [14]. It is responsible for processing tactile information such as touch, pressure, temperature, and pain.

Most SRS in the literature had been with the gamma knife, and only a handful on linac-based SRS for bAVM. While in metastatic brain lesions, GKS type of prescription to a lower isodose line at the periphery of the target may be beneficial since it gives a high dose to the central hypoxic region of the tumor, bAVMs on the other hand require full coverage of the optimal radiation dose for complete obliteration of the feeder vessels [16]. Furthermore, bAVMs tend to present in normal brain tissue/parenchyma, unlike brain metastasis, which usually pushes the brain parenchyma away. Hence, the extremely high dose of RT to the bAVM as with GKS, which can have a central dose in the range of 120 - 200% of the prescribed dose, may result in a higher brain necrosis rate, as seen in the recent publication comparing the toxicity of GKS versus linac-based SRS [17].

Modern computerized TPSs using inverse planning algorithms allow for multiple prescription dose levels and OAR constraint levels. For this patient, a volume prescription dose of 20Gy was chosen based on its effectiveness in obliterating bAVM. Prescription dose of above 23Gy did not result in significant benefit [18]. To minimize toxicity, a 14Gy prescription was selected near the adjacent OAR. With the computerized TPS, highly uniform dose coverage can be maintained inside the target, while intentional underdose can be delivered near critical structures or OARs (Figure 4). For OARs, some small part such as the surface of the brainstem can be allowed to receive a high dose, while the rest of the brainstem has a rigid constraint [19]. In this patient, the same concept was applied to the transverse sinus, whereby the medial inner wall of the sinus was allowed to receive 20Gy Dmax (maximum dose to the volume) and outer half of the transverse sinus was limited to 14Gy Dmax. The Dmax allowed to the cortical region controlling hand grip was 18Gy. We managed to achieve D0.2cc (maximum dose to 0.2cc of volume) of 17.6Gy and 13.4Gy to the hand grip cortex and medial wall of transverse sinus. Despite the constraints applied, the TPS managed to generate a treatment plan with 99% of the bAVM receiving 16.72Gy and 95% receiving nearly 20Gy (Table 1). The dose constraints for the OARs are not well defined in the literature, and we had to extrapolate the constraints from other structures in the brain such as carotid vessel and brain parenchyma, while keeping the high dose volume in the OAR to minimum [20].

The patient's temporary neurological deficit at three months is likely due to perilesional oedema rather than brain necrosis, explaining their full recovery and return to work. Furthermore, three months is too early for brain necrosis to occur after SRS. The small area of brain necrosis noted in the two-year post-treatment MRI is clinically not significant, and the patient is asymptomatic. Despite being in a very sensitive area, the minimal complications seen can be attributed to the volume-based prescription with VMAT technology and inverse planning algorithm in which the treating radiation oncologist can set the dose uniformity levels needed, avoiding extremely high dose regions within the target [19]. This is in contrast to GKS plans that typically prescribe peripheral doses to isodose levels ranging from 50% to 80%. The heterogeneity index in this patient is ≤1.6, indicating good dose homogeneity within the target. Although the dose conformity index for 20 Gy did not approach 1 (0.83 in this patient), this is expected due to the reduced prescription dose near the lateral sinus (Table 1).

The complete obliteration of the AVM feeder vessels as seen in this patient is the expected outcome in most patients by two years post treatment [6,7]. Since the bAVM is a benign condition and patients often survive long, it is essential to prevent or reduce long-term treatment complications. fMRI mapping of cortical areas and transmitting fiber pathways, combined with a complex treatment plan using advanced computerized TPS with inverse planning capability, aids in achieving this clinical outcome.

## Conclusions

With the current technological advent of linear accelerators and the TPS algorithms with inverse planning capacity, highly variable and conformal brain SRS plans can be generated based on individual patient’s disease anatomy. Since the bAVM is a benign disease and patients may have a long survival after treatment, it is important to ensure that the toxicity from the intervention is minimized. A multidisciplinary team successfully mapped adjacent brain areas, identified the bAVM target, and executed a complex radiotherapy plan, resulting in good outcomes for a patient with a parietal bAVM near the hand grip region and left lateral sinus.

## References

[1] Derdeyn CP, Zipfel GJ, Albuquerque FC, Cooke DL, Feldmann E, Sheehan JP, Torner JC (2017). Management of brain arteriovenous malformations: a scientific statement for healthcare professionals from the American Heart Association/American Stroke Association. Stroke.

[2] Al-Shahi R, Warlow C (2001). A systematic review of the frequency and prognosis of arteriovenous malformations of the brain in adults. Brain.

[3] Mohr JP, Overbey JR, Hartmann A (2020). Medical management with interventional therapy versus medical management alone for unruptured brain arteriovenous malformations (ARUBA): final follow-up of a multicentre, non-blinded, randomised controlled trial. The. Lancet Neurol.

[4] Kim H, Al-Shahi Salman R, McCulloch CE, Stapf C, Young WL (2014). Untreated brain arteriovenous malformation: patient-level meta-analysis of hemorrhage predictors. Neurology.

[5] Lv X, Wu Z, Li Y, Yang X, Jiang C (2012). Hemorrhage risk after partial endovascular NBCA and ONYX embolization for brain arteriovenous malformation. Neurol Res.

[6] Bollet MA, Anxionnat R, Buchheit I (2004). Efficacy and morbidity of arc-therapy radiosurgery for cerebral arteriovenous malformations: a comparison with the natural history. Int J Radiat Oncol Biol Phys.

[7] Chang JH, Chang JW, Park YG, Chung SS (2000). Factors related to complete occlusion of arteriovenous malformations after gamma knife radiosurgery. J Neurosurg.

[8] Provenzi L, Lindstedt J, De Coen K (2021). The paternal brain in action: a review of human fathers’ fMRI brain responses to child-related stimuli. Brain Sci.

[9] Chang Mulato JE, Kus WP, Biondi Soares LG (2022). Microsurgical treatment for arteriovenous malformation in eloquent area: a 2-dimensional surgical video and step-by-step practical guide. World Neurosurg.

[10] Puckett A, Panchuelo R (2023). Imaging somatosensory cortex: human functional magnetic resonance imaging. Somatosensory Research Methods.

[11] Corniani G, Saal HP (2020). Tactile innervation densities across the whole body. J Neurophysiol.

[12] Zoeller AC, Drewing K (2020). A systematic comparison of perceptual performance in softness discrimination with different fingers. Atten Percept Psychophys.

[13] Arikan BE, Voudouris D, Voudouri-Gertz H, Sommer J, Fiehler K (2021). Reach-relevant somatosensory signals modulate activity in the tactile suppression network. Neuroimage.

[14] Seri FA, Hamid AA, Abdullah J, Zamzuri I, Omar H (2019). Brain responses to frequency changes due to vibratory stimulation of human fingertips: An fMRI study. J Phys Conf Ser.

[15] Tomasino B, Bernardis P, Maieron M, D'Agostini S, Skrap M (2023). Parietal/premotor lesions effects on visuomotor cognition in neuro-oncology patients: a multimodal study. Neuropsychologia.

[16] Wu N, Wang Z, Guo X, Zhao H (2023). Dose-effect relationship of linear accelerator based stereotactic radiotherapy for brain metastases. Radiat Oncol.

[17] Dharnipragada R, Dusenbery K, Watanabe Y, Ferreira C, Chen CC (2024). Comparison of Gamma Knife (GK) and Linear Accelerator (LINAC) radiosurgery of brain metastasis resection cavity: a systematic review and proportional meta-analysis. Clin Exp Metastasis.

[18] Flickinger JC, Kondziolka D, Maitz AH, Lunsford LD (2002). An analysis of the dose-response for arteriovenous malformation radiosurgery and other factors affecting obliteration. Radiother Oncol.

[19] Wang D, DeNittis A, Hu Y (2020). Strategies to optimize stereotactic radiosurgery plans for brain tumors with volumetric-modulated arc therapy. J Appl Clin Med Phys.

[20] Milano MT, Usuki KY, Walter KA, Clark D, Schell MC (2011). Stereotactic radiosurgery and hypofractionated stereotactic radiotherapy: normal tissue dose constraints of the central nervous system. Cancer Treat Rev.

